# External Quality Assessment (EQA) program for the preanalytical and analytical immunohistochemical determination of HER2 in breast cancer: an experience on a regional scale

**DOI:** 10.1186/1756-9966-32-58

**Published:** 2013-08-21

**Authors:** Irene Terrenato, Vincenzo Arena, Sara Pizzamiglio, Ilaria Pennacchia, Letizia Perracchio, Simonetta Buglioni, Cristiana Ercolani, Francesca Sperati, Leopoldo Costarelli, Elena Bonanno, Daniela Baldini, Silvia Candia, Anna Crescenzi, Antonella Dal Mas, Claudio Di Cristofano, Vito Gomes, Lucia Rosalba Grillo, Paola Pasquini, Maria Nicoletta Pericoli, Maria Teresa Ramieri, Domenica Di Stefano, Luigi Ruco, Stefania Scarpino, Domenico Vitolo, Giulia d’Amati, Angelo Paradiso, Paolo Verderio, Marcella Mottolese

**Affiliations:** 1 Biostatistic Unit, Regina Elena National Cancer Institute, Via Elio Chianesi 53, Rome, Italy; 2 Unit of Medical Statistics, Biometry and Bioinformatics, Fondazione IRCCS Istituto Nazionale dei Tumori, Milan, Italy; 3 Institute of Pathology, Catholic University of Sacred Heart, Largo Agostino Gemelli 8, Rome, Italy; 4 Pathology Department, Regina Elena National Cancer Institute, Via Elio Chianesi 53, Rome, 00144, Italy; 5 Department of Pathology, San Giovanni-Addolorata Hospital, Via dell’Amba Aradam 9, Rome, Italy; 6 Anatomic Pathology, Dept of Biomedicine and Prevention University of Rome Tor Vergata, Viale Oxford 81, Rome, Italy; 7 Pathology Department, San Filippo Neri Hospital, Via Martinotti 20, Rome, Italy; 8 Pathology Department, Sandro Pertini Hospital, Via dei Monti Tiburtini 385, Rome, Italy; 9 Pathology Department, Regina Apostolorum Hospital, Via S.Francesco 50, Albano Laziale, Rome, Italy; 10 Pathology Department, San Salvatore Hospital, Via Vetoio-Coppito, L’Aquila, Italy; 11 Department of Experimental Medicine, Sapienza University of Rome, I.C.O.T, Via Franco Faggiana 68, Latina, Italy; 12 Pathology Department, Bel Colle Hospital, Strada S. Martinese, Viterbo, Italy; 13 Pathology Department, San Camillo Forlanini Hospital, Via Gianicolense 1, Rome, Italy; 14 Pathology Department, Coelio Military Hospital, Piazza Cellimontana 50, Rome, Italy; 15 Pathology Department, S.Maria Goretti Hospital, Via Guido Reni 1, Latina, Italy; 16 Pathology Department, ASL Frosinone, Viale Mazzini 94, Frosinone, Italy; 17 Department of Cytology and Histology, University of Rome La Sapienza, Ospedale S. Andrea, Via di Grottarossa 1035, Rome, 00189, Italy; 18 Department of Radiological, Oncological and Pathological Sciences, Sapienza University of Rome, Policlinico Umberto I, Viale Regina Elena 324, Rome, 00161, Italy; 19 Clinical Experimental Oncology Department, National Cancer Research Centre, Istituto Tumori Giovanni Paolo II, Bari, Italy

**Keywords:** Breast Neoplasm/drug therapy, Receptor erbB-2/analysis, Immunohistochemistry/methods, Quality control, Reproducibility of results

## Abstract

**Background:**

An External Quality Assessment (EQA) program was developed to investigate the state of the art of HER2 immunohistochemical determination in breast cancer (BC) in 16 Pathology Departments in the Lazio Region (Italy). This program was implemented through two specific steps to evaluate HER2 staining (step 1) and interpretation (step 2) reproducibility among participants.

**Methods:**

The management activities of this EQA program were assigned to the Coordinating Center (CC), the Revising Centers (RCs) and the Participating Centers (PCs). In step 1, 4 BC sections, selected by RCs, were stained by each PC using their own procedures. In step 2, each PC interpreted HER2 score in 10 BC sections stained by the CC. The concordance pattern was evaluated by using the kappa category-specific statistic and/or the weighted kappa statistic with the corresponding 95% Jackknife confidence interval.

**Results:**

In step 1, a substantial/almost perfect agreement was reached between the PCs for scores 0 and 3+ whereas a moderate and fair agreement was observed for scores 1+ and 2+, respectively.

In step 2, a fully satisfactory agreement was observed for 6 out of the 16 PCs and a quite satisfactory agreement was obtained for the remaining 10 PCs.

**Conclusions:**

Our findings highlight that in the whole HER2 evaluation process the two intermediate categories, scores 1+ and 2+, are less reproducible than scores 0 and 3+. These findings are relevant in clinical practice where the choice of treatment is based on HER2 positivity, suggesting the need to share evaluation procedures within laboratories and implement educational programs.

## Background

HER2 is one of the most important therapeutic targets in breast cancer (BC). Trastuzumab, the humanized anti-HER2 monoclonal antibody (MoAb), that specifically binds the extracellular domain of the protein, is a drug that, in combination with different chemotherapy regimens, has sensibly modified the survival of patients with HER2 positive BC. In addition, the introduction of other novel anti HER2 treatments [1] such as lapatinib [2], pertuzumab [3] and T-DM1 [4], just shows how increasingly important it is to correctly identify BC patients who may benefit from these target therapies. Therefore, it is the pathologist’s responsibility to assure accurate HER2 determination and reliable results in BC and beyond BC [5,6]. Along with the different methods used in routine clinical practice, the most common, extensively validated by international guidelines [7], are immunohistochemistry (IHC) and fluorescent (FISH) or chromogenic (CISH/SISH) in situ- hybridization. For most of the prospective randomized adjuvant trials of trastuzumab, testing algorithms for HER2 mainly consisted in initial IHC followed by ISH for equivocal score 2+ [8]. Despite the fact that trastuzumab is considered the drug for excellence in HER2 positive metastatic [9,10], locally advanced and early BC [8], diagnostic approaches to assess the HER2 status are often vital and the need to solve many controversial issues in oncogene testing still pose a challenge [11,12]. The reliability of the IHC assay is affected by several sources of variability which depends on a considerable number of factors, both analytical, pre-analytical and interpretative that may influence the final results. The latest guidelines drafted by the American Society of Clinical Oncology and the College of American Pathologists highlighted that up to now 15% to 20% [7] of current HER2 testing are inaccurate thus, significantly affecting therapeutic decision making. In the U.S.A. [13] and Great Britain [14,15], the UK National External Quality Assessment Scheme (NEQAS) defined the minimum quality criteria to which the pathologist has to adhere to guarantee a valid process of specific biomarker determinations, both for prognostic and predictive markers. Within these criteria, it is foreseen to participate in external quality control assessment (EQA) programs. In the last ten years, the Italian Network for Quality Assessment of Tumor biomarkers (INQAT) promoted and implemented several EQA studies [16]. INQAT activities, relating to the evaluation of the performance level of participant laboratories in IHC determination of HER2 in BC, initially focused its attention on evaluating the analytical phase (interpretation) of the biomarker [17], then gradually extended into the pre-analytical phase (tissue staining) [18]. In this context, we decided to conduct a two-step EQA study involving 16 pathology laboratories in the Lazio Region in Italy in order to evaluate their performance related to both the staining (step1) and the interpretation (step2) of IHC HER2 assay. The overall purpose of the study is to provide shared solutions to the common problems that may routinely occur during the biomarker determination process. The present paper reports the results of this regional EQA program.

## Methods

### Study design

The management activities of this EQA program were assigned to different working units: the Coordinating Center (CC), the Revising Centers (RCs) and the Participating Centers (PCs). The CC, that coordinated the logistical and practical aspects of the EQA, collected a series of HER2 positive and negative BC cases from its own archive. A group of three reviewers (RCs), chosen based on their expertise in terms of the high number of HER2 tests per year, together with a pathologist of the CC, contributed in selecting the BC slides to be included in the EQA and in defining the HER2 IHC score to be used as reference value. In a detailed protocol, written before the start of the program, the aim of the study, the study design, the criteria for the selection of the cases, the HER2 evaluation procedure according to the ASCO-CAP guidelines [7] and the statistical analysis strategy were described. All 16 pathology laboratories that agreed to participate in the study accepted the protocol and filled out a questionnaire before the start of the study in order to gather information regarding their routine methods in the HER2 determination.

The primary aim of this EQA consisted in evaluating the performance of each participant in relation to the whole process of HER2 determination. For this purpose, the EQA program was implemented via two specific steps: EQA HER2 immunostaining and EQA HER2 interpretation.

**Figure 1:**
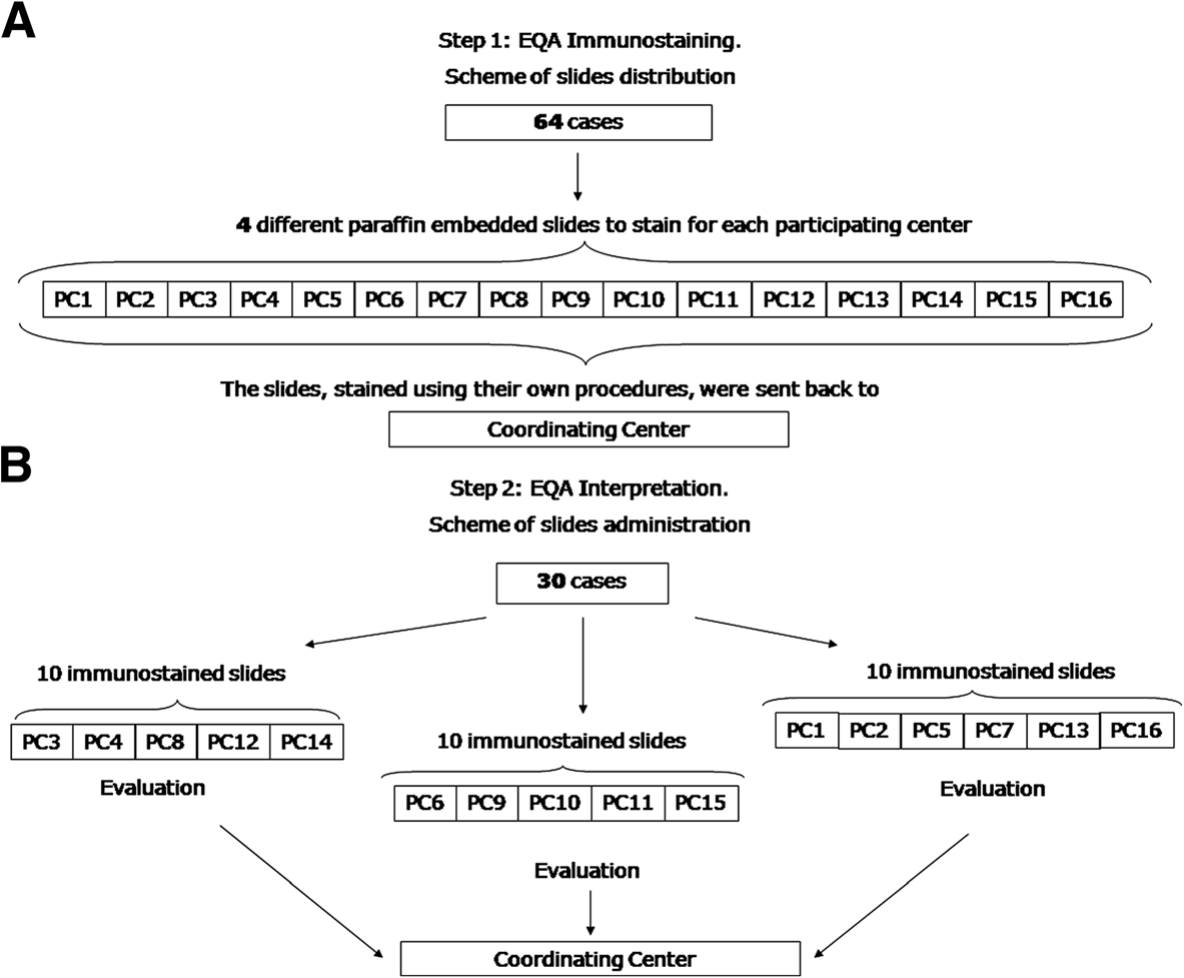
**Workflow of the EQA program. A**. EQA HER2 immunostaining: specimens were selected and sent by the Coordinating Center (CC) to the 16 PCs. **B**. EQA HER2 interpretation: specimens were selected and sent by the CC to the 16 PCs grouped into 3 sets.

In the EQA HER2 immunostaining step, 64 BC cases were selected and each PC received 4 different BC sections. The PCs stained the slides by adopting their own procedures that were previously reported in the questionnaire and then sent them back to the CC (Figure 1A). The interpretation of all the 64 slides was performed by the group of RCs. For the EQA HER2 interpretation step, the 16 PCs were randomly divided into three groups. A set of 10 slides, for a total of 30 different BC cases, rotated among the participants belonging to each group (Figure 1B). Each set was generated in such a way as to fully cover the range of HER2 values usually observed in routine practice in order to include an adequate number of slides with intermediate scores (1+; 2+). Three out of the 16 score 2+ (19%) BC resulted amplified by SISH test. Each participant interpreted the HER2 IHC score according to the ASCO-CAP guidelines [7].

The study was reviewed and approved by the Ethics Committee of the Regina Elena National Cancer Institute and a signed informed consent was obtained from all patients.

### Statistics

In the EQA HER2 immunostaining step, the performance of each laboratory was evaluated by comparing the reviewer’s interpretation of the slides stained by each laboratory according to the reference values. In addition, in order to evaluate the contribution of each scoring category to the overall agreement (i.e. the agreement between the score given by the reviewers on the slides stained by each laboratory in accordance with the reference values) the kappa category-specific (k_cs_) statistic [19], and its 95% confidence interval obtained by means of the Jackknife method [20], were calculated as previously described [21,22]. To this end, the slides stained by all the participants were jointly considered. Each k_cs_ value was interpreted in a qualitative manner based on the Landis and Koch classification criteria [23].

In the EQA HER2 interpretation step, the level of agreement of each laboratory according to the reference values was evaluated by computing the weighted kappa statistic (k_w_) and its 95% Jackknife confidence interval as previously described. In line with our previous experience with EQA programs, the agreement was considered fully satisfactory only when the lower limit of the 95% Jackknife confidence interval was equal to or greater than 0.80. For each participant the k_cs_ statistic and its 95% Jackknife confidence interval were also computed. Statistical analyses were performed with the SAS software (Version 9.2.; SAS Institute Inc., Cary, NC).

## Results

### Questionnaire

The results of the questionnaire are reported in Table 1. Frequency distribution of the responses indicates moderate methodological heterogeneity between the 16 laboratories. All the PCs used paraffin embedded tissue and the DAB chromogen in their routine. Most PCs adopted buffered formalin during fixation. Twenty-four hours was the modal fixation time and also the modal time elapsing between cutting to IHC. For more than two thirds of participants, the slides were stored at room temperature. Only 5 PCs used the manual immunostaining procedure. The polyclonal antibody A0485 purchased by Dako was the most commonly used reagent. The majority of PCs used a heat retrieval in an automated immunostainer. Only one participant used an image analyzer for evaluating the sample in addition to the optical microscope in their routine. For about two thirds of the participants the criteria used to evaluate the samples considered the percentage of positive cells. Furthermore, the different number of samples assayed per year reflects the different level of experience among the PCs.

**Table 1:** Questionnaire results from the 16 participant centers

	**N (%)**
**N° annual HER2 determination**	
<100	4 (25%)
100-500	10 (62.5%)
>500	2 (12.5%)
**Material**	
Paraffin embedded tissue	16 (100%)
**Type of fixation**	
Buffered formalin	12 (75%)
Formalin	2 (12.5%)
Other	2 (12.5%)
**Time of tissue fixation**	
12 hours	2 (12.5%)
24 hours	10 (62.5%)
Other	4 (25%)
**Time from cutting to IHC**	
24 hours	10 (62.5%)
1 week	4 (25%)
other	2 (12.5%)
**Slide storage**	
37°C	5 (31%)
Room temperature	11 (69%)
**Immunostaining procedure**	
Automated	11 (69%)
Manual	5 (31%)
**Type of reagent**	
A0485 PoAb	11 (69%)
CB11 MoAb	4 (25%)
Herceptest	1 (6%)
**Chromogen**	
DAB	16 (100%)
**Evaluation**	
Optical microscope	15 (94%)
Optical microscope + Image analyzer	1 (6%)
**Evaluation criteria**	
Score and % of positive cells	10 (62.5%)
Score	6 (27.5%)

### EQA HER2 immunostaining

In regards to EQA HER2 immunostaining, Table 2 shows the frequency of misclassifications observed in relation to the reference score in the 64 cases studied. For 5 PCs all the slides were correctly immunostained. Six PCs provided 3 out of 4 slides in accordance with the reference value. For the remaining 5 PCs the correspondence between their score and reference value was found for 2 out of 4 slides.

**Table 2:** HER2 immunostaining: misclassifications in relation to the reference score

**ID**	**Total N° of misclassified slides**^**(#)**^	**Reference score 0**^**(#)**^	**Reference score 1 +** ^**(#)**^	**Reference score 2 +** ^**(#)**^	**Reference score 3 +** ^**(#)**^
PC1	0/4	- (*)	0/1	0/1	0/2
PC2	1/4	0/1	0/1	0/1	**1/1 [2+]**
PC3	1/4	0/1	**1/2 [0]**^**^**^	- (*)	0/1
PC4	2/4	0/1	**1/1 [0]**	**1/1 [1+]**	0/1
PC5	2/4	0/1	- (*)	**1/1 [1+]**	**1/2 [2+]**
PC6	1/4	0/1	0/1	**1/1 [1+]**	0/1
PC7	2/4	0/2	- (*)	- (*)	**2/2 [2+;2+]**
PC8	0/4	- (*)	0/2	0/1	0/1
PC9	2/4	0/1	**2/2 [0;0]**	- (*)	0/1
PC10	1/4	0/2	- (*)	**1/1 [1+]**	0/1
PC11	1/4	0/1	0/1	**1/1 [1+]**	0/1
PC12	0/4	0/1	0/2	- (*)	0/1
PC13	0/4	0/1	- (*)	0/1	0/2
PC14	0/4	- (*)	0/2	0/1	0/1
PC15	1/4	0/1	**1/2 [0]**	- (*)	0/1
PC16	2/4	0/1	**1/1 [2+]**	**1/1 [1+]**	0/1
**Total**	**16/64**	**0/15**	**6/18**	**6/11**	**4/20**

(*) Score not received. ^(#)^N° of misclassified slides/N° of received slides. ^ Brackets report the score provided by PCs.

All the PCs gave a correct immunostaining concerning score 0. Six immunostained slides did not correspond to the reference score 1+: among these, five slides were given a score of 0 and one a 2+ score. Concerning score 2+, six slides were not immunostained correctly and all of them were given a score of 1+. Finally, concerning score 3+, four slides were not immunostained properly and all of them were given a score of 2+. Due to a problem of logistics not all the participants received one slide per HER2 score.

Table 3 reports the kappa category-specific statistic values (k_cs_) and the relative 95% Jackknife confidence interval to indicate how each scoring category contributed to the agreement overall. As expected, the categories with a lower level of reproducibility are the two intermediate ones (score 1+ and score 2+). In particular, when we used the Landis-Kock classification criteria as a measure of agreement, the score of 1+ versus the reference score was “moderate” (with a value between 0.41 to 0.60), while for the score 2+ the agreement was “fair” (with a value between 0.21 to 0.40). In the other two categories, score 0 and 3+, the agreement was substantial /almost perfect (greater than 0.80).

**Table 3:** **k**_**cs **_**statistic and 95% Jackknife confidence interval by HER2 score**

**Score**	**N slides**	**k**_**cs**_	**95% Confidence interval of k**_**cs**_	
			**Lower limit**	**Upper limit**
0	64	0.80	0.64	0.97
1+	64	0.54	0.31	0.78
2+	64	0.37	0.07	0.70
3+	64	0.85	0.70	1.00

### EQA HER2 interpretation

Table 4 summarizes the results obtained from the EQA HER2 interpretation step. Only two PCs provided scores equal to reference ones for all the 10 slides. Four PCs provided one discordant value out of 10, misclassifying the reference value score 1+ in three cases and score 2+ in one case. It is worthy to note, that no score 3+ was misclassified and only 1 score 0 was interpreted as score 1+. Conversely, we observed 12 and 14 misclassifications in score 1+ and 2+, respectively.

**Table 4:** HER2 interpretation: misclassifications in relation to the reference score

**ID**	**Group**	**Total N° of misclassified slides**^**(#)**^	**Reference score 0**^**(#)**^	**Reference score 1 +** ^**(#)**^	**Reference score 2 +** ^**(#)**^	**Reference score 3 +** ^**(#)**^
PC1	3	1/10	0/2	**1/3 [2+]**	0/3	0/2
PC2	3	2/10	0/2	0/3	**2/3 [1+;1+]**	0/2
PC3	1	1/10	0/2	**1/3**^(*)^	0/3	0/2
PC4	1	2/10	0/2	0/3	**2/3 [1+;1+]**	0/2
PC5	3	0/10	0/2	0/3	0/3	0/2
PC6	2	2/10	0/2	**1/3 [2+]**	**1/3 [3+]**	0/2
PC7	3	0/10	0/2	0/3	0/3	0/2
PC8	1	2/10	**1/2 [1+]**^**^**^	0/3	**1/3 [1+]**	0/2
PC9	2	1/10	0/2	**1/3 [2+]**	0/3	0/2
PC10	2	2/10	0/2	**1/3 [2+]**	**1/3 [1+]**	0/2
PC11	2	2/10	0/2	**1/3 [2+]**	**1/3 [1+]**	0/2
PC12	1	2/10	0/2	**1/3 [2+]**	**1/3 [1+]**	0/2
PC13	3	3/10	0/2	**2/3 [2+;2+]**	**1/3 [1+]**	0/2
PC14	1	1/10	0/2	0/3	**1/3 [1+]**	0/2
PC15	2	2/10	0/2	**1/3 [2+]**	**1/3 [3+]**	0/2
PC16	3	4/10	0/2	**2/3 [0;0]**	**2/3 [1+;1+]**	0/2
**Total**	**27/160**	**1/32**	**12/48**	**14/48**	**0/32**	

(*) Slide not evaluated. ^(#)^N° of misclassified slides/N° of received slides. ^Brackets report the score provided by PCs.

Table 5 shows the k_w_ values and the relative lower limit of the 95% confidence interval obtained by comparing the scores provided by PCs with the reference values. Overall, by considering the point-estimate values of the k_w_ statistic a satisfactory agreement was reached between the reference score and the one provided by the evaluation of each PC. However, by taking into account the lower limits of the 95% confidence interval of the k_w_ statistic, only 6 PCs reach a fully satisfactory agreement.

**Table 5:** **k**_**w **_**statistic values between the reference scores and scores reported by PCs**

**ID**	**Group**	**k**_**w**_	**k**_**w **_**95% CI**	
			**Lower limit**	**Upper limit**
PC1*	3	0.95	0.86	1.00
PC2	3	0.90	0.76	1.00
PC3*	1	1.00	1.00	1.00
PC4	1	0.90	0.76	1.00
PC5*	3	1.00	1.00	1.00
PC6	2	0.91	0.80	1.00
PC7*	3	1.00	1.00	1.00
PC8	1	0.89	0.72	1.00
PC9*	2	0.95	0.86	1.00
PC10	2	0.90	0.77	1.00
PC11	2	0.90	0.77	1.00
PC12	1	0.90	0.77	1.00
PC13	3	0.87	0.71	1.00
PC14*	1	0.95	0.86	1.00
PC15	2	0.91	0.80	1.00
PC16	3	0.84	0.67	1.00

(*) These PCs reached a fully satisfactory agreement.

Table 6 reports the distribution of the k_cs_ statistics (and relative 95% confidence interval) obtained by comparing each PC with the reference value. From this table it emerges that the two most problematic categories are the middle ones, score 1+ and score 2+. In particular, score 2+ reached a moderate agreement (the median-value is between 0.41 and 0.60) while score 1+ reached a substantial agreement (the median-value is between 0.61 and 0.80). In the other two categories, the agreement, represented by its median value, resulted perfect.

**Table 6:** **Minimum, median and maximum of k**_**cs **_**statistic distribution versus the reference score**

	**Score 0**	**Score 1+**	**Score 2+**	**Score 3+**
**Minimum**	0.54	0.05	0.35	0.74
**Median**	1.00	0.67	0.52	1.00
**Maximum**	1.00	1.00	1.00	1.00

## Discussion

During these years it has become increasingly important to constantly verify, through national and international quality control studies, the performance of pathology laboratories in biomarker determinations, especially the ones that aim to identify those patients eligible for treatment with targeted therapies.

An accurate and reproducible detection of HER2 protein overexpression and/or gene amplification plays a key role in determining the future course of BC treatment, especially in the light of recent data which have demonstrated promising clinical efficacy of novel biological agents, such as the anti-HER2 MoAbs Pertuzumab and TDM1 [3,4]. However, the accuracy and interlaboratory reproducibility of HER2-status assessment is still a worldwide concern [16-18]. It is significantly crucial to define and follow fundamental steps in the conduction of quality control studies in order to minimize the potential bias in reproducing the two intermediate classes, namely 1+ and 2+ scores.

Our two-step EQA study was carried out in a community clinical practice setting on regional scale which allowed to evaluate the whole process of IHC HER2 determination. This program was not designed to be formative, but its informative nature gave an important overview of the state of the art of HER2 determination in the Lazio region.

This EQA program stresses the need of rigorous quality-control procedures for preparing and analysing breast tumors specimens. It also provided interesting results that confirm those of previous quality control programs of HER2 testing [24]. In particular, the observed agreement showed a good level of standardization of HER2 determination procedures within each laboratory for scores of 0 and 3+ (both for the immunostaining and the interpretation phases) but revealed a low degree of reproducibility of the two intermediate scoring classes (1+ and 2+).

Among the 6 misclassifications observed in relation to reference score 1+, one was 2+ which erroneously identified a potentially eligible patient for trastuzumab therapy. Six out of 11 cases with score 2+ were misclassified as 1+ excluding potentially eligible patients from the correct therapy regimen. Conversely, the 4 score 3+ cases, classified as 2+, would probably lead the pathologist to look for HER2 gene amplification.

The latter results represent what routinely happens in pathology laboratories and may explain why a few breast cancer cases classified positive for HER2 do not really respond to anti-HER2 therapy. Another important issue, as recently reported [25], is the modulation of HER2 status between primary and metastatic tumors. This discordance may be imputable to technical limitations in HER2 testing which may not be simply due to the increasing level of genetic instability occurring throughout disease progression. Several aspects related to both pre-analytical and analytical phase, may have led to not achieving completely satisfactory results due to differences in tissue fixation times, reagents and immunohistochemistry protocols. Discordant results mostly occur in borderline positive samples, emphasizing the level of subjectivity in HER2 evaluation in reproducing the intermediate scoring categories. These data are in line with other literature on EQA studies [24,26] and support the conclusion that the definition of shared procedures may overcome these limitations by providing more consistent and reproducible diagnostic results.

## Conclusions

In summary, the results of our EQA program showed that diagnostic approaches in assessing the HER2 status are often essential. In fact, we observed a good level of standardization of HER2 determination procedures within each laboratory for scores 0 and 3+. Conversely, a low degree of reproducibility for score 1+ and 2+ was found. In this context, it is obvious that there is a need to solve these controversial issues in oncogene testing through implementing EQA programs.

We strongly believe that EQA programs, focused on the whole process of HER2 testing performed on a regional scale, should be promoted on a national scale. Participation in these programs may provide a tool for improving the performance level even in experienced laboratories.

## Competing interests

The authors declare that they have no competing interests.

## Authors’ contribution

IT, VA, PV and MM contributed equally to this study. IT, SP and PV: study design, statistical analysis, data interpretation and paper writing; IP, AP data interpretation and paper writing; FS and CE: data collection and interpretation; MM, VA, LC, EB: immunohistochemistry performance and interpretation, paper writing. LP, SB, DB, SC, AC, AD, CDC, VG, LRG, PP, MNP, MTR, DDS, LR, SS, DV, GD: immunohistochemistry performance. All authors read and approved the final manuscript.
